# FLAIR vascular hyperintensity combined with asymmetrical prominent veins in acute anterior circulation ischemic stroke: prediction of collateral circulation and clinical outcome

**DOI:** 10.1186/s40001-023-01445-4

**Published:** 2023-10-19

**Authors:** Wei Xiang, Hongchun Wei, Zhigang Liang, Manman Zhang, Zhongwen Sun, Yaodong Lv, Chengzhou Zhang, Huaguang Zheng

**Affiliations:** 1 Department of Neurology, Yantai Yuhuangding Hospital Affiliated to Qingdao University, No. 20 East Yuhuangding Road, Yantai, 264000 Shandong China; 2 Yantai Regional Sub-Center of China National Clinical Research Center for Neurological Diseases, Yantai, China; 3 The Second Clinical Medical College, Binzhou Medical University, Yantai, China; 4 Department of Radiology, Yantai Yuhuangding Hospital Affiliated to Qingdao University, Yantai, China; 5 Department of Neurology, Beijing Tiantan Hospital, Capital Medical University, Beijing, China

**Keywords:** Acute ischemic stroke, Asymmetrical prominent veins sign, Susceptibility-weighted imaging, FLAIR vascular hyperintensity, Collateral circulation, Prognosis

## Abstract

**Background:**

To investigate the value of fluid-attenuated inversion recovery vascular hyperintensity (FVH) within asymmetrical prominent veins sign (APVS) on susceptibility-weighted imaging predicting collateral circulation and prognosis in patients with acute anterior circulation ischemic stroke.

**Method:**

Patients with severe stenosis or occlusion of ICA or MCA M1, who underwent MRI within 72 h from stroke onset were reviewed. The Alberta Stroke Program Early CT Score was used to evaluate the volume of infarction on DWI, the degree of FVH and APVS. Spearman correlation analysis was used to evaluate the correlation between FVH and APVS. All patients were divided into the good prognosis group and the poor prognosis group according to the score of the modified ranking scale (mRS) 90 days after the stroke. Logistic regression analysis was used to explore the relationship between FVH and APVS and functional prognosis, while receiver operating characteristic (ROC) curves were plotted to assess the value of FVH and APVS in predicting prognosis.

**Results:**

Spearman correlation analysis revealed moderate positive correlations between FVH and APVS (*r* = 0.586, *P* < 0.001). The poor prognosis group had a higher rate of a history of atrial fibrillation, a larger cerebral infarction volume, a higher NIHSS score at admission, and a higher FVH and APVS score compared with the good prognosis group (all *P* < 0.05). A further logistic regression indicated that the NIHSS score, cerebral infarction volume, FVH and APVS were independent risk factors for a poor functional prognosis. In terms of FVH, APVS, alone and their combination for the diagnosis of poor prognosis, the sensitivity, specificity, area under the ROC curve (AUC), and 95% confidence interval (CI) were 86.8%, 83.3%, 0.899 (95% CI 0.830–0.968); 60.5%, 93.7%, 0.818 (95% CI 0.723–0.912); 86.8%, 89.6%, 0.921 (95% CI 0.860–0.981), respectively.

**Conclusion:**

The presence of FVH and APVS can provide a comprehensive assessment of collateral circulation from the perspective of veins and arteries, and the correlation between the two is positively correlated. Both of them were independent risk factors for poor prognosis, their combination is complementary and can improve the predictive value.

## Introduction

Acute ischemic stroke (AIS) has a large burden of morbidity and mortality worldwide, accounting for 87% of all total strokes [1]. Identification of irreversible infarct core and salvageable penumbra is essential to guide the therapeutic decision. The clinical practice found that there was a mismatch between the volume of infarction and the therapeutic effects, as well as the functional prognosis. This diversity may be partly explained by the different compensatory capacities of collateral circulation [2, 3]. Good collaterals enable timely reperfusion of blood flow to the ischemic penumbra, including reducing the volume of cerebral infarction, prolonging the therapeutic window, decreasing neurological impairments, and improving prognosis [4]. Recently, imaging techniques with contrast media have been introduced for the evaluation of collateral circulation, however, considering the complication of irreversible renal damage caused by contrast injection, and the inability to perform it in patients with renal insufficiency or contrast medium allergy, it is necessary to find a more non-invasive imaging method to estimate collaterals [5].

Fluid-attenuated inversion recovery (FLAIR) is now routinely used to improve the detection of lesions in patients with AIS because of its ability to suppress cerebrospinal fluid hyper-signal. FLAIR vascular hyperintensity (FVH) was probably due to compensatory reflux from the distal to the proximal of a stenotic or occluded vessel, as the slow blood flow would lead to a loss of the flowing void effect and result in hyperintensity, serving as a non-contrast visualization marker of leptomeningeal collaterals [6–8]. When the primary collateral circulation cannot provide enough blood perfusion, the secondary and tertiary collateral vessels will be established. Among them, the opening of leptomeningeal collaterals is an important pathway. FVH could serve as an objective basis for the formation of leptomeningeal collateral circulation after stroke [9]. Many studies have confirmed that FVH was correlated with circulation as well as the severity of hemodynamic impairments, opening up a new idea of exploring noninvasive assessment of the status of intracranial collaterals and prognosis [10–12].

Susceptibility-weighted imaging (SWI) is a novel, high-resolution, three-dimensional gradient-echo MR technique, characterized by being highly sensitive to paramagnetic substances and has been implemented in the assessment of cerebral hemodynamics following AIS [13]. Asymmetrical dilated-vessel-like signal loss seen in the ischemic cerebral hemisphere on SWI, namely asymmetrical prominent veins sign (APVS), can indirectly show an increase of oxygen extraction fraction (OEF) in the penumbral, has been increasingly recognized as an alternative to assess collaterals and an imaging biomarker for predicting prognosis [14–16]. It has been widely believed that the APVS correlated with uncoupling between the oxygen supply and demand in the hypoperfusion tissue, resulting in a relative increase in deoxyhemoglobin [17]. To our knowledge, no studies have been found on exploring the correlation between the combination of FVH and APVS in the evaluation of collateral circulation in AIS, and predicting the functional prognosis. Therefore, this study aimed to investigate the relationship between FVH, APVS and prognosis, to explore the correlation between the status of collaterals and relevant factors affecting prognosis, and to provide an imaging basis for the blood perfusion status in the ischemic area, individualized precise treatment and prognosis evaluation.

## Methods

### Participants

We performed a retrospective study of patients with AIS hospitalized in the Affiliated Yantai Yuhuangding Hospital of Qingdao University between July 1, 2021 and October 1, 2022. Patients were eligible if they met the following inclusion criteria: (i) age ≥ 18 years; (ii) diagnosis of anterior circulation stroke due to occlusion or server stenosis (> 70%) of the unilateral internal carotid artery (ICA) and/or M1 segment of the middle cerebral artery (MCA); (iii) multimodal MR protocol including diffusion-weighted imaging (DWI), fluid-attenuated inversion recovery (FLAIR), time-of-flight MR angiography (TOF-MRA) and SWI sequence was performed within 72 h after symptom onset; and (iv) pre-stroke modified Rankin scale score ≤ 1; The exclusion criteria were (i) MRI-confirmed of the posterior circulation or bilateral infarction; (ii) absence of complete clinical and imaging data; (iii) intracranial hemorrhage evident on CT imaging; and (iv) patients who underwent intravenous or intra-arterial thrombolytic therapy.

This study protocol was approved by the institutional review board of our hospital. All subjects provided informed consent in accordance with the Declaration of Helsinki.

### Collection of clinical data

Demographic characteristics (age and sex) and clinical characteristics, including vascular risk factors (hypertension, diabetes, dyslipidemia, ischemic heart disease, atrial fibrillation, prior stroke/transient ischemic attack (TIA), active smoking and drinking history), the admission National Institute of Health Stroke Scale (NIHSS) score was collected from electronic medical records. All patients received antiplatelet or anticoagulation therapy as the guideline recommended [18]. Clinical outcomes were assessed with the modified Rankin Scale (mRS) at 90 days after stroke onset. Good and poor functional outcomes were defined by the mRS scores of 0–2 and 3–5, respectively [19].

### MRI protocol

Multimodal MRI was performed using a 3.0-T system (GE Discovery 750, USA). The imaging protocol included T1 (time repetition (TR) = 1750 ms, time echo (TE) = 24 ms, field of view (FOV) = 220 mm, slice thickness = 5 mm, matrix = 320 × 320, duration = 1 min 51 s), T2 (TR = 5392 ms, TE = 120 ms, Matrix = 240 × 240, FOV = 240 mm, section thickness = 5 mm, duration = 1 min 21 s), DWI(TR = 5237 ms, TE = 101 ms, b-value = 1000 s/mm^2^, FOV = 240 mm, section thickness = 5 mm, section gap = 1.5 mm, matrix = 240 × 240, acquisition duration = 42 s), FLAIR(TR = 9000 ms; TE = 100 ms; flip angle = 16°; matrix = 300 × 240; section thickness = 5 mm; acquisition duration = 1 min 57 s;), three-dimensional time-of-flight magnetic resonance angiography (TOF-MRA) (TR = 20 ms; TE = 2.5 ms; flip angle = 20°; matrix = 272 × 240; section thickness = 1.4 mm; acquisition duration = 5 min 10 s) and SWI (TR = 86.1 ms, TE = 45 ms, Matrix = 267 × 240, flip angle = 15°, FOV = 240 mm, section thickness = 2.0 mm, duration = 5 min 40 s).

### Image analysis

One experienced neuroradiologist and one trained neurologist, blinded to the clinical parameters of the patients, assessed all MR imaging independently. Any discrepancies were resolved and consensus. The volume of infarction on DWI images was assessed by the standard Alberta Stroke Program Early CT score (DWI-ASPECTS). Fresh infarcts were visible in each region with a score of 1. The total score ranges from 10 (complete ischemic involving the whole MCA territory) to 0 (without early ischemic change) [20].

**Fig. 1:**
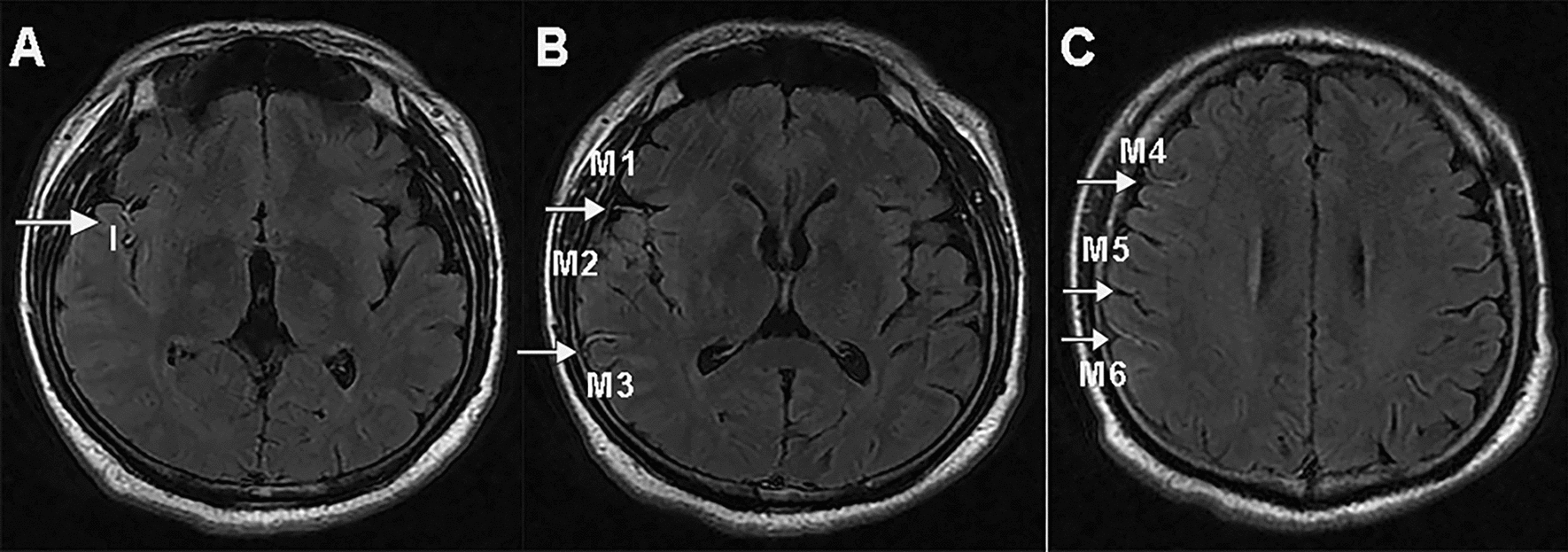
Images illustrating FVH: blood supply in area of the left MCA (M1, M2, M3, Insular, M4, M5, M6) showing FVH and FVH-ASPECTSTS score was 7; I, insular cortex; M1, the anterior MCA cortex, corresponded with the frontal operculum; M2, the MCA cortex lateral to the insular ribbon, corresponded with the anterior temporal lobe; M3, the posterior MCA cortex, corresponded with the posterior temporal lobe. M4, M5, and M6, the anterior, lateral, and posterior MCA territories immediately superior to M1, M2, and M3, respectively

The FHV was defined as linear or serpentine high signal intensity relative to gray matter along the cortical sulci or brain surface in the cerebral hemisphere, reflecting the status of leptomeningeal collaterals [21]. The FVH-ASPECTS [22], a scoring system ranging following insular and M1-M6 regions was used to quantify the FVH (Fig. 1). The presence of FVH in each region was scored as 1.0 indicating the absence of FVH and 7 suggesting prominent FVH.

**Fig. 2:**
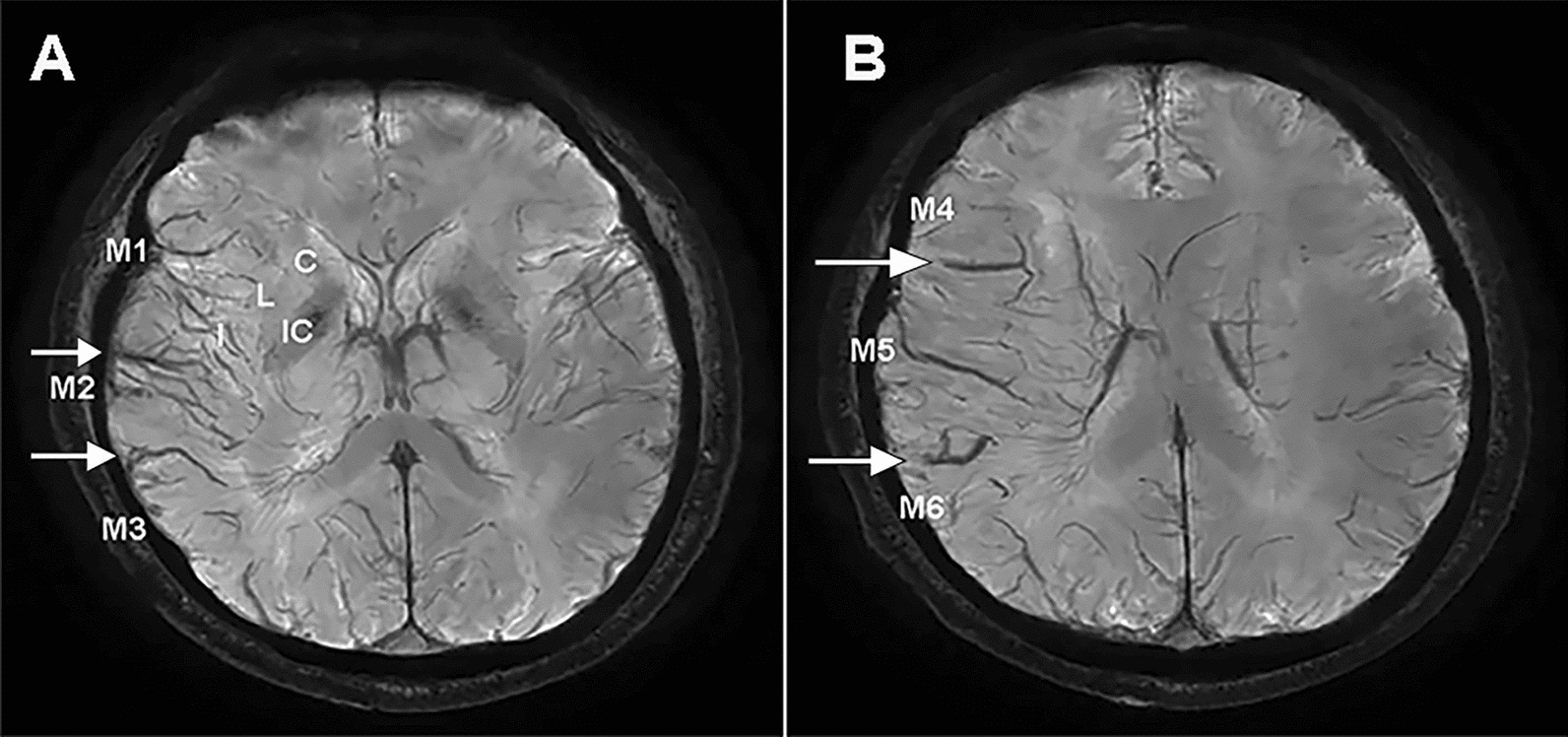
Example of APVS-ASPECTS score. A typical extensive APVS image which are visible on seven areas of the middle cerebral artery (MCA) territory (M1, M2, M3, Insula, M4, M5, M6) and the APVS-ASPECTS score was 7

APVS was defined as a prominence of hypointense vessels with either more numerous or larger veins and greater signal loss in the ischemic target compared with the contralateral hemisphere [23, 24]. The ASPECTS score was used to assess the extent of APVS [25, 26]. The MCA territory was divided into ten areas (Caudate nucleus, Lentiform nucleus, Internal capsule, Insular, M1–M6) (Fig. 2). A score on a scale of 0–10 was given, with 0 being none APVS and 10 being APVS was presented in all areas of the MCA.

### Statistical analysis

The data were analyzed using SPSS v.24.0. All metric and normally distributed variables were reported as means and standard deviations (SD), and non-parametric distributed variables were described as medians and interquartile ranges (IQR). Categorical variables were presented as frequencies and percentages. The student *t*-test was used to compare normally distributed continuous variables; the Mann–Whitney *U* test was used when continuous variables failed tests for normality; the chi-square test or Fisher’s exact test for categorical variables as appropriate. Inter-observer agreement was assessed using the intraclass correlation coefficient (ICC). Spearman’s correlation was used to assess the correlation between the FVH score and the APVS score. The good and poor prognosis were analyzed by univariate analysis. Then all variables with *P* < 0.1 were included in the multivariable analysis. Multivariable logistic regression analysis was performed to identify independent factors associated with poor prognosis. The receiver operating characteristic (ROC) curve was used to analyze the predictive value of FVH, APVS and their combination in assessing the poor prognosis. *P*-values < 0.05 were considered statistically significant.

## Results

### Patient demographics and variables’ correlation analysis

**Fig. 3:**
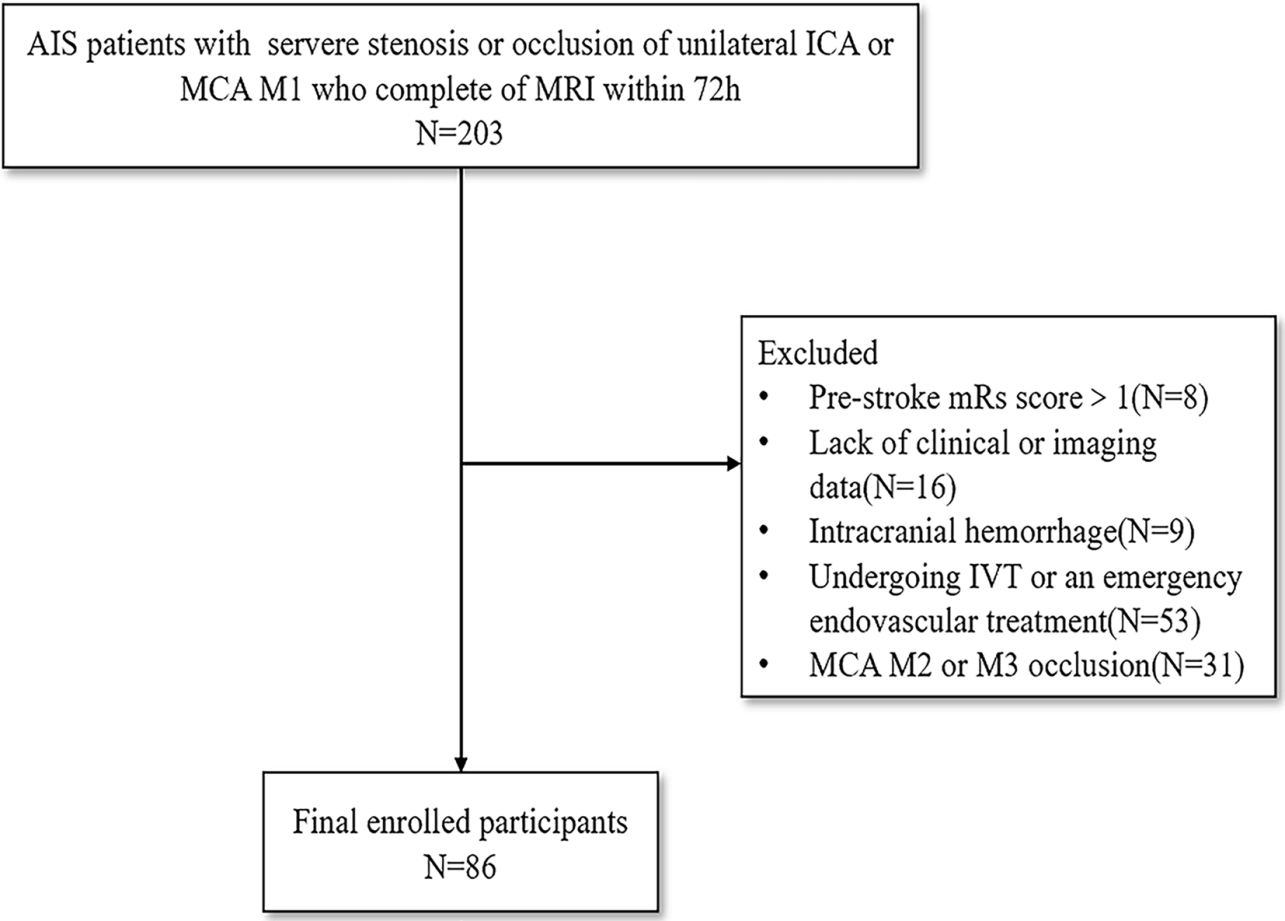
Flow diagram of enrollment of study patients

A total of 203 patients were assessed for study eligibility and 86 patients were enrolled in this study (Fig. 3). The mean age was 66.14 ± 11.39 years. The overall rate of FVH presence was 86.0%, and the rate of APVS presence was 70.9%. The media FVH-ASPECTS was 3.0 (interquartile range [IQR], 2.0–5.0), the media APVS-ASPECTS was 4.0 (IQR, 1.0–6.0). The media of DWI-ASPECTS score was 4.0 (IQR, 3.0–6.0), the median admission NIHSS score was 8.5 (IQR, 5.0–12.0), and the median time from onset to MRI imaging was 42.0 h (IQR, 31.0–53.3).

**Fig. 4:**
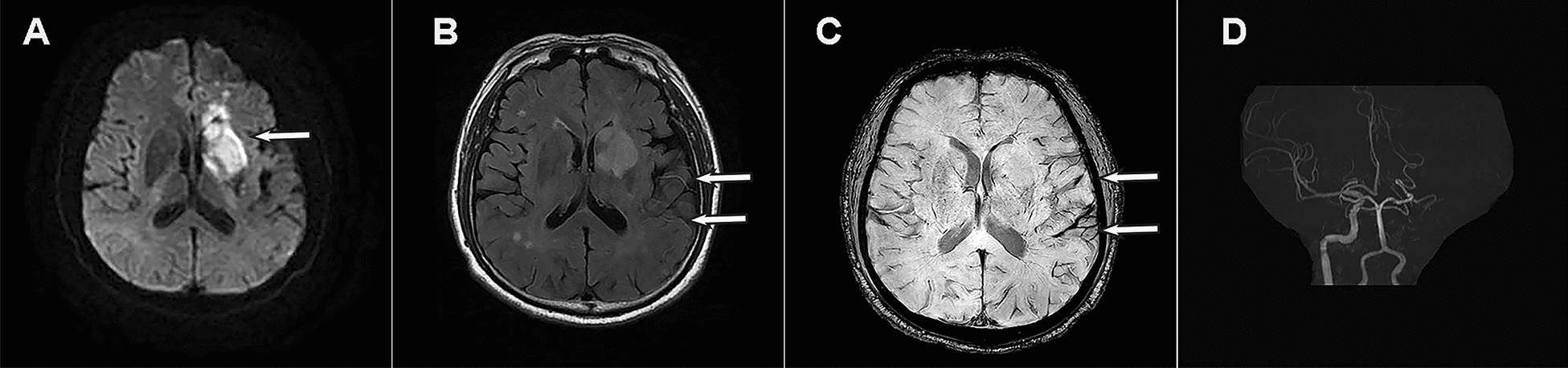
Presentation of a typical patient. A 66-year-old man with left limb weakness was admitted to the hospital, with a 30-h between onset to MR examination. **A** DWI showed acute infarction; **B** FLIAR image demonstrated vascular hyperintensity (FVH); **C** SWI indicated asymmetrical prominent veins sign (APVS); **D** MRA demonstrated disappearance of left ICA and MCA signals

The quality of MRI images of enrolled patients met the diagnostic standards and allowed to identify FVH and APVS. The agreement between readers was excellent for FVH on FLAIR images (ICC = 0.982) and for APVS on SWI (ICC = 0.951). Scatter plots showed a moderate positive correlation between FVH-ASPECTS and APVS-ASPECTS (*r* = 0.586, *P* < 0.001). Figure 4 shows typical serial images.

### Factors associated with prognosis

Patients with poor prognosis had a higher rate of atrial fibrillation history, a significantly higher admission NIHSS score, larger cerebral infarction volume, higher APVS-ASPECTS and FVH-ASPECTS score than patients with good prognosis (all *P* < 0.05). There were no significant differences between the groups in age, sex, stroke risk factors (hypertension, diabetes mellitus, hyperlipidemia, ischemic heart disease, previous stroke or TIA, active smoking and drinking), MRI time from stroke onset and the vascular site and degree of steno-occlusion. The details are presented in Table 1.

**Table 1:** Comparisons of clinical and imaging characteristics in patients with good and poor outcome

Characteristics	Good outcome (*n* = 48)	Poor outcome (*n* = 38)	*P*-value
Age at onset, mean ± SD	65.60 ± 10.82	66.82 ± 12.19	0.627
Male, *n* (%)	24(50.0)	26(68.4)	0.085
Medical history, *n* (%)			
Hypertension	33(68.8)	23(60.5)	0.427
Diabetes mellitus	13(27.1)	9(23.7)	0.720
Hyperlipidemia	14(29.2)	10(26.3)	0.770
Ischemic heart disease	7(14.6)	2(5.3)	0.161
Atrial fibrillation	6(12.5)	13(34.2)	0.016
Previous stroke or TIA	12(25.0)	5(13.2)	0.171
Drinking	8(16.7)	11(28.9)	0.173
Smoking	15(31.3)	17(44.7)	0.199
Time to MRI examination, median (IQR)	40.5(31.0–53.0)	43.5(30.0–54.0)	0.913
NIHSS score at admission, median (IQR)	6.0(4.0–8.0)	12.0(9.0–16.0)	< 0.001
DWI-ASPECTS, median (IQR)	3.0(2.0–4.0)	6.0(4.8–7.3)	< 0.001
Site of steno-occlusion, *n* (%)			0.224
ICA or M1 severe stenosis	13(27.1)	8(21.1)	
ICA occlusion	12(25.0)	4(10.5)	
M1 occlusion	14(29.2)	17(44.7)	
ICA and M1 occlusion	9(18.8)	9(23.7)	
APVS-ASPECTS	6.0(4.0–7.0)	2.0(0–4.0)	< 0.001
FVH-ASPECTS	5.0(4.0–6.0)	2.0(0.3–3.0)	< 0.001

NIHSS, National Institute of Health Stroke Scale; DWI, Diffusion weighted imaging; ASPECTS, Alberta Stroke Program Early CT Score; ICA, internal carotid artery; M1, M1 segment of middle cerebral artery; APVS, asymmetrical prominent veins sign; FVH, Fluid-attenuated inversion recovery vascular hyperintensity

Multivariable logistic regression analysis showed that FVH-ASPECTS (Odd ratio (OR) 2.485, 95% confidence interval (CI) 1.145–5.390, *P* = 0.021), APVS-ASPECTS [OR 1.801; 95% CI 1.094–2.965; *P* = 0.021], admission NIHSS score (OR 1.869, 95% CI 1.107–3.155, *P* = 0.019) and DWI-ASPECTS score (OR 3.104, 95% CI 1.104–8.730, *P* = 0.032) were independently associated with a poor prognosis (Table 2**)**.

**Table 2:** Multivariate logistic regression of risk factors for predicting poor outcome

Variable	Odds ratio	95% CI	*P*-value
Atrial fibrillation	3.611	0.291–44.726	0.317
Admission NIHSS	1.869	1.107–3.155	0.019
DWI-ASPECTS	3.104	1.104–8.730	0.032
APVS-ASPECTS	1.801	1.094–2.965	0.021
FVH-ASPECTS	2.485	1.145–5.390	0.021

ASPECTS, Alberta Stroke Program Early CT Score; APVS, asymmetrical prominent veins sign; FVH, Fluid-attenuated inversion recovery vascular hyperintensity; CI, confidence interval

**Fig. 5:**
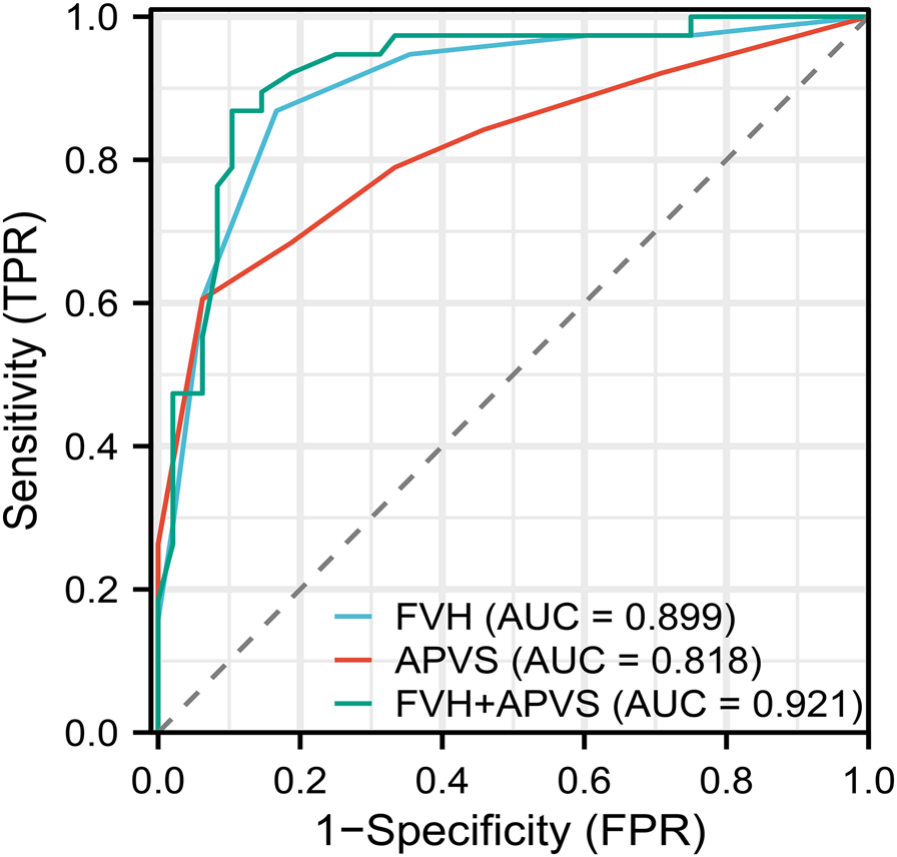
The ROC curve of FVH and APVS alone and their combination to predict a poor outcome. ROC, receiver operating characteristic; FVH, FLAIR vascular hyperintensity; APVS, asymmetrical prominent veins sign

The ROC curves for FVH and APVS individually and their combination for predicting poor prognosis are presented in Fig. 5. Interestingly, the area under the curve (AUC) of FVH was 0.899 (95% CI 0.830, 0.968), the sensitivity was 0.868, the specificity was 0.833. While the AUC of APVS was 0.818 (95% CI 0.723, 0.912), with a sensitivity was 0.605, and a specificity was 0.937. Moreover, the AUC of their combination was 0.921(95% CI 0.860, 0.981, *P* < 0.001), and the sensitivity and specificity to predict poor prognosis were 86.8% and 89.6% respectively. The FVH, APVS and their combination with an optimal cut-off 0.459, 0.665, and 0.452, respectively (Table 3).

**Table 3:** Areas under the ROC curves for each parameter of FVH and APVS individually and their combination for predicting poor prognosis

Parameters	Cut-off	Sensitivity	Specificity	AUC	95% CI	*P*-value
FVH	0.459	0.868	0.833	0.899	0.830–0.968	< 0.01
APVS	0.665	0.605	0.937	0.818	0.723–0.912	< 0.01
FVH + APVS	0.452	0.868	0.896	0.921	0.860–0.981	< 0.01

AUC, area under the curve; APVS, asymmetrical prominent veins sign; FVH, Fluid-attenuated inversion recovery vascular hyperintensity; CI, confidence interval

## Discussion

Our results showed a positive correlation between the FVH-ASPECTS and APVS-ASPECTS, suggesting that the presence of extensive FVH and APVS in the acute phase of stroke can both reflect the status of collaterals. We also found that the significant predictors of a poor prognosis in AIS treated without recanalization due to occlusion or stenosis of the unilateral ICA and/or M1 segment of the MCA within 72 h of symptom onset were higher baseline NIHSS scores, larger baseline DWI lesion volume, more extensive FVH and APVS grading. In addition, the combination of the FVH and APVS was more predictive of poor prognosis than either alone.

Good collateral flow is increasingly recognized as a crucial element in therapeutic and prognostic assessment, as it can markedly reduce penumbra loss and prolong the salvaged time [27]. The new concept of collateral histology reveals that collateral circulation includes the entire cerebral circulatory system including the arterial, microvascular and venous systems. Consequently, collateral vessels can be judged indirectly via capillary horizontal perfusion, metabolic brain tissue status and changes in intracranial drainage venous pressure besides direct visual imaging technology [28]. FVH and APVS can reflect the hypoperfusion state of damaged brain tissue in patients with AIS and provide information on blood perfusion for assessing the status of the collateral circulation. This study used the ASPECTS score system to quantify the extent of FVH and APVS, and analyzed the correlation between them on this basis. Regarding FVH and APVS, previous studies reached a consensus about their mechanism that patients with severe stenosis or occlusion of large vessels were more likely to have extensive FVH and APVS [15, 29]. When the infarction caused by severe stenosis or occlusion of large vessels occurred, brain tissue in the infarct core area experienced a sequence of pathophysiological mechanisms, including ischemia, hypoxia, cell edema and so on. Under the stimulation of ischemia and hypoxia, the establishment and opening of the leptomeningeal collaterals occurred, along with changes in brain oxygen metabolism and vein expansion, resulting in an increase in oxygen uptake fraction to maintain the basic metabolic level of brain cells. Our study also demonstrated a positive correlation between FVH score and APVS score, which further confirmed the hemodynamic mechanisms of FVH and APVS from both arterial and venous perspectives. Despite the distinct physiological mechanisms of FVH and APVS, they both can indicate inadequate perfusion of brain tissue and reflect the status of collateral circulation in the acute phase.

In clinical practice, research has shown a higher incidence of disability in individuals with anterior cerebral circulation stroke. Subsequently, the early identification of useful prognostic markers plays a critical role in treatment selection and timely intervention. Our study discovered that patients with poor outcomes exhibited increased occurrences of atrial fibrillation in their medical history, higher NHISS scores at baseline, larger baseline infarct volumes, and more extensive FVH and APVS. We suggested that a history of atrial fibrillation is associated with a poor prognosis, possibly due to the abrupt onset of stroke in patients with cardiogenic embolism, the lack of time for collateral branches to form around the ischemic tissue and insufficient compensation of collaterals can result in more severe hypoperfusion [30]. This also highlights the significance of distinguishing stroke subtypes caused by different pathological mechanisms for predicting functional prognosis. The NIHHS score is a widely-used scale for assessing the severity of neurological deficits and plays a crucial role in predicting their functional prognosis. Generally, higher NIHHS scores indicate greater neurological impairment and poorer functional outcomes, and vice versa. A further logistic regression analysis showed that the NIHSS score was an independent risk factor for a poor prognosis. The volume of infarction strongly predicted clinical prognosis has been reported earlier [31]. Our study similarly identified an independent correlation between infarct volume and poor prognosis in AIS patients. The larger the irreversible damage volume of brain tissue, the more severe the clinical dysfunction and unfavorable recovery of the ischemic semidark zone, and the worse the prognosis.

Currently, research on the relationship between FVH and functional outcomes varies with time. The duration of time from symptom onset to imaging has been reported as an essential factor for evaluating the prognostic value of FVH [32]. In most studies that suggested a correlation between FVH and favorable outcomes, the time from symptom onset to imaging was less than 6 h [33–35]. In comparison, in the majority of studies that demonstrated an association between FVH and unfavorable outcomes, the symptom-to-imaging time was 12–24 h or longer [36–38]. Our findings confirmed that FVH was independently associated with poor prognosis, which we believe is related to the fact that the patients we enrolled who did not receive timely vessel recanalization and underwent MR scanning within 72 h. Previous studies believed that FVH may serve as an imaging marker of leptomeningeal collaterals within 6 h of symptom onset or within the time window of reperfusion treatment [39]. The extent of FVH may indicate the amount of brain tissue vulnerable to ischemia, which can be reversed with reperfusion therapy to reduce the size of the eventual lesion and improve functional outcomes. This clarifies why FVH-positive patients tend to have superior clinical outcomes compared to those FVH-negative patients during this specific time interval. However, when FVH appears beyond the time window for reperfusion therapy, it may indicate persistent occlusion of the vessel and hypoperfusion. Thus, patients with FVH may be more susceptible to hemodynamic instability than patients with and without FVH. This variability might be related to the severity of clinical impairment and unfavorable outcomes in patients with FVH during this period. We recommended further investigation into the potential mechanisms underlying this difference.

APVS was found to be another independent factor associated with a 90-day poor prognosis in our study. To some extent, extensive APVS represents an acute ischemic penumbra with poor perfusion. As the hypoperfusion time increases, the size of the ischemic penumbra diminishes, which is predictive of early neurological deterioration (END) and stroke progression [16]. Several studies have investigated the correlation between APVS and prognosis. Some argued that APVS was not associated with prognosis [40, 41], while others believed that patients with APVS had a worse prognosis than those patients without APVS [26, 42, 43]. Chen et al. [40] considered that extensive APVS was associated with poor prognosis, whereas patients without APVS tended to have better collaterals compensation and no obvious hypoperfusion area, thus making it easier to obtain a good prognosis. Sun et al. [44] demonstrated that APVS was a predictor of poor prognosis and was independently associated with END. In our study, patients with extensive APVS had a worse functional prognosis, possibly due to the absence of thrombolytic treatment. Conservative therapy was primarily given to individuals with contraindications to thrombolytic or over a time-window, with more underlying conditions. Although collateral circulation existed at this time, it was more susceptible to stroke progression due to the inability to receive timely and effectively. As a result, it carries a higher overall probability of resulting in a poor prognosis [45]. This outcome may provide clinicians with novel insights into the precise management of stroke, as timely endovascular therapy within the time window is crucial for improving prognosis. We further clarified the value and efficacy of FVH and APVS alone and their combination in predicting poor outcome by ROC curves. The combination AUC was higher than that of each individual index and can compensate for the limitations of the evaluation of each individual index. These findings also offered a degree of reference for the clinical treatment assessment and functional prognosis prediction.

There are several limitations in this study. It is a retrospective analysis and has a potential risk of selection bias, without analysis of patients who underwent thrombolysis or endovascular therapy. Infarct volume determined by ASPECTS is influenced by the quality of the images and this may lead to bias; Finally, FVH and APVS were not observed dynamically in this study, a longitudinal study investigating the association with the change of FVH and APVS and the progression of collateral as well as prognosis is needed.

## Conclusions

When stroke occurred, apart from the rapid establishment and opening of the leptomeningeal collaterals in the ischemia and hypoxia area, there is also an increase in the oxygen uptake fraction of brain tissue to co-compensate for the ischemic and hypoxic state. The presence of FVH and APVS can comprehensively assess collateral circulation from both the vein and artery perspective, and there is a positive correlation between the two. Besides, the two indicators were independently related to a poor prognosis at 90 days, and their combination can enhance the predictive accuracy. Using the FLAIR and SWI sequence together is useful for an early non-invasive assessment of collateral status, the selection of individualized treatment and improves the prediction of clinical outcome. Further studies with a larger sample size are needed to confirm our results.

## Data Availability

Data are available upon reasonable request. The data that support the findings of this study are available from the corresponding author, upon reasonable request.
